# Cul4-Ddb1 ubiquitin ligases facilitate DNA replication-coupled sister chromatid cohesion through regulation of cohesin acetyltransferase Esco2

**DOI:** 10.1371/journal.pgen.1007685

**Published:** 2019-02-19

**Authors:** Haitao Sun, Jiaxin Zhang, Siyu Xin, Meiqian Jiang, Jingjing Zhang, Zhen Li, Qinhong Cao, Huiqiang Lou

**Affiliations:** Beijing Advanced Innovation Center for Food Nutrition and Human Health and State Key Laboratory of Agro-Biotechnology, College of Biological Sciences, China Agricultural University, Beijing, China; The University of North Carolina at Chapel Hill, UNITED STATES

## Abstract

Cohesin acetyltransferases ESCO1 and ESCO2 play a vital role in establishing sister chromatid cohesion. How ESCO1 and ESCO2 are controlled in a DNA replication-coupled manner remains unclear in higher eukaryotes. Here we show a critical role of CUL4-RING ligases (CRL4s) in cohesion establishment via regulating ESCO2 in human cells. Depletion of CUL4A, CUL4B or DDB1 subunits substantially reduces the normal cohesion efficiency. We also show that MMS22L, a vertebrate ortholog of yeast Mms22, is one of DDB1 and CUL4-associated factors (DCAFs) involved in cohesion. Several lines of evidence show selective interaction of CRL4s with ESCO2 through LxG motif, which is lost in ESCO1. Depletion of either CRL4s or ESCO2 causes a defect in SMC3 acetylation, which can be rescued by HDAC8 inhibition. More importantly, both CRL4s and PCNA act as mediators for efficiently stabilizing ESCO2 on chromatin and catalyzing SMC3 acetylation. Taken together, we propose an evolutionarily conserved mechanism in which CRL4s and PCNA promote ESCO2-dependent establishment of sister chromatid cohesion.

## Author summary

During the cycle of cell division and proliferation, each chromosome is copied into twin sister chromatids. To make sure a complete set of chromosomes are correctly passed on from generation to generation, the twins must be tethered together by a multi-protein ring called cohesin. ESCO1 and ESCO2 have been known to catalyze the acetylation of a cohesin subunit SMC3, which triggers the establishment of sister chromatid cohesion. Here, we have shown that CUL4-DDB1 ubiquitin ligases (CRL4^MMS22L^), in collaboration with PCNA, promote this key reaction. CRL4s selectively bind and stabilize ESCO2 on chromatin through a particular motif, which is lost in its cousin ESCO1. This explains the functional divergence and division of labor between these two paralogs. Both CRL4^MMS22L^ and PCNA are known components of the moving DNA replication machines. So, our results help us to understand how twin sister chromatids become cohesive concomitantly with chromosome duplication process in human cells.

## Introduction

Faithful genetic inheritance requires precise chromatin replication and separation of sister chromatids into two daughter cells. To ensure accurate chromosome segregation in eukaryotic cells, each pair of sister chromatids must be aligned properly and held together by a cohesin complex from S phase to anaphase [1–6]. The cohesin complex is a four-subunit ring conserved from yeast to human. In human mitotic cells, cohesin is composed of SMC1, SMC3, RAD21 (Scc1/Mcd1 in yeast) and SA1 or SA2 (Scc3 in yeast) [2, 7–10].

Cohesin is widely believed to have distinct statuses according to its association with chromatin during the cell cycle. In G_1_ phase, it is loaded loosely onto chromatin (i.e., non-cohesive status) [11]. As cells proceed into S phase, cohesin binds more tightly to hold sister chromatids together (i.e. cohesive status). This transition is called the establishment of sister chromatid cohesion [5, 12]. Although the structural bases of this transition remain enigmatic, it has been shown to depend on an essential cohesin acetyltransferase, Eco1 in yeast (*Saccharomyces cerevisiae*) [13–15]. Eco1, whose essential substrate is proved to be Smc3 [13], triggers the cohesion establishment during S phase through counteracting the opposing activity of Rad61 (WAPL in human) [16].

Cohesion is established in a DNA replication-coupled manner [12, 17, 18]. To achieve this, the activity of Eco1 is controlled concomitantly with DNA replication through two independent mechanisms. First, Eco1 contains a canonical PIP (PCNA interaction protein) box, which mediates its interaction with PCNA, the multivalent-platform of DNA replisome [19]. Second, a member of the cullin-RING E3 ligases (CRLs) Rtt101-Mms1, associates with the replication fork and facilitates Smc3 acetylation via direct association between Eco1 and the substrate receptor component of the ligase, Mms22 [20].

CRLs constitute the largest ubiquitin ligase family in eukaryotes. They are modular assemblies consisting of a Cullin scaffold in complex with an adapter and distinct ligase substrate receptors, giving rise to many combinatorial possibilities. There are three cullins in budding yeast (Cul1, 3 and 8) and eight in human (Cul1, 2, 3, 4A, 4B, 5, 7 and 9) [21]. Cul8, also known as Rtt101, is unique to budding yeast, yet it shows low sequence similarity with CUL4. Nevertheless, the Rtt101 adaptor Mms1 is highly homologous to human DDB1 adapter of CUL4, and Rtt101-Mms1 performs similar functions to CUL4-DDB1 ligases in other species [22]. The human genome encodes two CUL4 paralogs, CUL4A and CUL4B, which share 80% sequence identity aside from CUL4B having an extended N-terminus containing a nuclear localization signal (NLS) [23]. Both CUL4A and CUL4B use DDB1 as an adaptor and DCAFs (DDB1 and CUL4-associated factors) as substrate receptors to recognize a large number of substrate proteins [24–26]. CRL4s play recognized roles in DNA repair, replication and chromatin modifications through ubiquitylation and/or mediating protein-protein interactions [27, 28].

Both of the two Eco1 orthologs in mammalian cells, ESCO1 and ESCO2 [29], have been shown to acetylate SMC3 at two evolutionarily conserved lysine residues (K105K106) [15, 30, 31]. Interestingly, ESCO1 acetylates SMC3 through a mechanism distinct from that of ESCO2 [32]. Nevertheless, how the activities of ESCO1 and ESCO2 are controlled to establish replication-coupled sister chromatid cohesion in vertebrates has not been delineated.

In this study, we report that CUL4-DDB1 E3 ligases participate in establishing sister chromatid cohesion in human cells. Depletion of CUL4A, CUL4B or DDB1 results in precocious sister chromatid separation in 293T cells. We show that MMS22L (Mms22-like), the human ortholog of yeast Mms22, the substrate receptor of Rtt101-Mms1, interacts with DDB1. Interestingly, ESCO2, not ESCO1, co-immunoprecipitates with all CRL4^MMS22L^ subunits. Dosage suppression experiments reveal that CRL4s and ESCO2 are able to compensate each other in SMC3 acetylation and thereby sister chromatid cohesion. Through introducing interaction-defective mutations, we find that ESCO2 acetylates SMC3 dependent on interactions with both CUL4-DDB1 ligases and PCNA. These suggest that CUL4-DDB1 ligases and PCNA contribute together to connect ESCO2-dependent cohesion establishment with the replication process in human.

## Results

### CUL4-DDB1 and MMS22L are required for efficient sister chromatid cohesion in human cells

**Fig 1:**
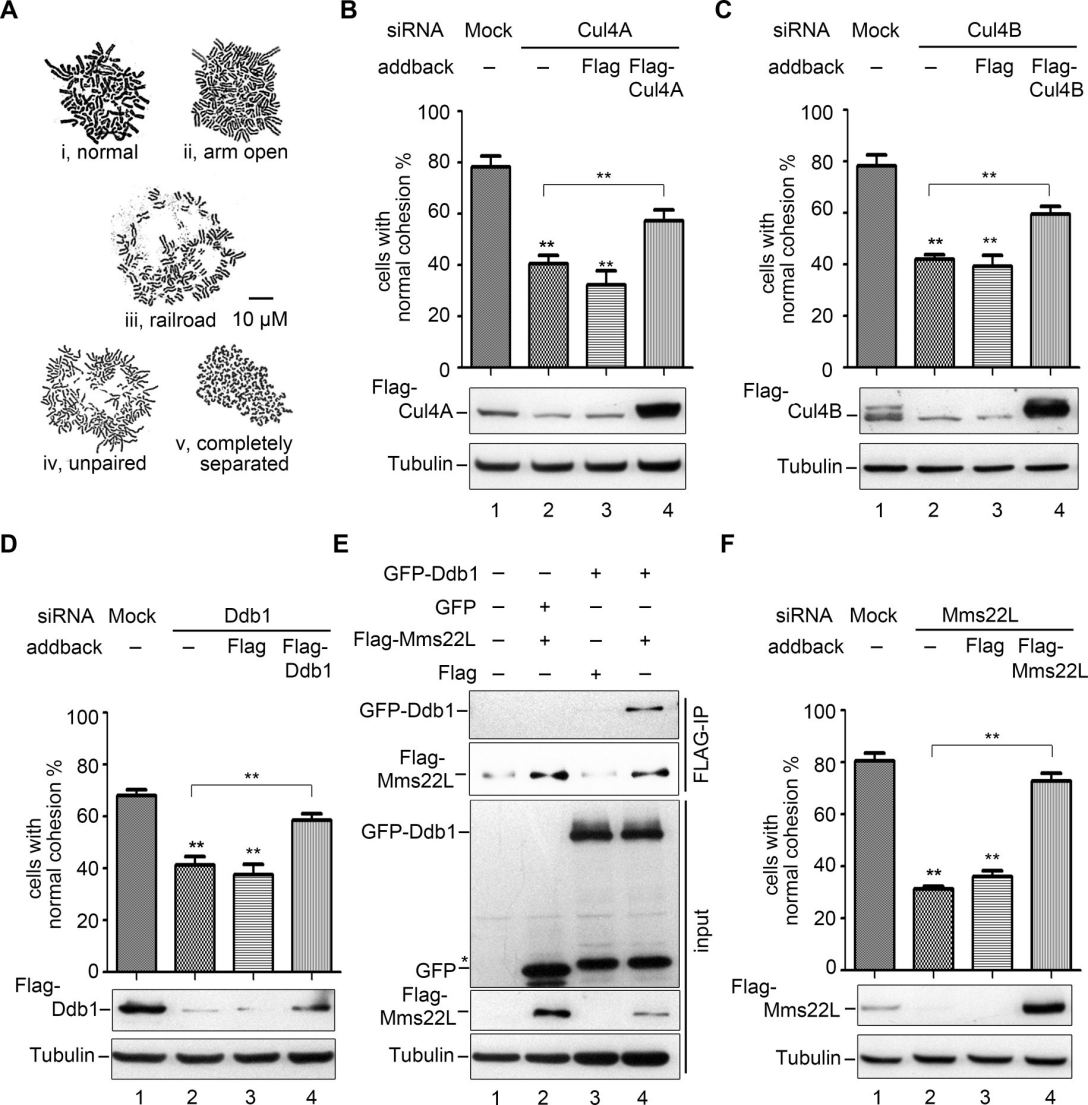
Knockdown of CUL4-DDB1-MMS22L causes severe cohesion defects. (A) Representative morphologies of human chromosome spreads stained with Giemsa. Closed sister chromatids (i) indicate normal cohesion, while loose sister chromatids (arms open, loosely paired, unpaired and completely separated, ii-v) indicate different extents of cohesion defects. We have only rarely observed that chromosomes within one cell display different morphologies. In order to reflect the physiological status of chromosome morphologies, cells were not synchronized. At least 200 mitotic cells were enriched and harvested via trypsin digestion for each experiment. The percentage of cells with closed sister chromatids among the total mitotic cells (i.e., normal cohesion %) was quantified from at least three independent experiments. (B-D) Cohesion defects caused by depletion of CUL4A (B), CUL4B (C) and DDB1 (D). 293T cells were transfected with siRNAs specific to CUL4A, CUL4B and DDB1 for 48 h. Plasmids expressing the indicated Flag-tagged protein were transferred into the cells after 6 h for the complementation assay. The trysinized cells were fixed with methanol and acetic acid (3:1) for three times and then stained with Giemsa. More than 200 mitotic cells per RNAi experiment were scored; the results of at least three independent biological experiments were summarized in the histogram. The cohesion percentage of each RNAi sample was compared with that of mock or add-back using student’s *t*-test, **P<0.01. The efficiency of siRNA and complementation of CUL4A, CUL4B, DDB1 or MMS22L was detected via immunoblots against the indicated antibodies. (E) DDB1 co-precipitates with MMS22L. *Flag*, *Flag-MMS22L* and *GFP*, *GFP-DDB1* plasmids were transferred into 293T cells. After IP experiments, MMS22L and DDB1 were detected with antibodies against Flag and GFP, respectively. Tubulin was probed as a loading control. (F) MMS22L depletion leads to compromised cohesion as well. Quantification of the cohesion percentage was performed as described above. See S1 Fig for the *CEN* cohesion defect results.

Recently, we showed that fork-associated Rtt101-Mms1 ubiquitin ligases take part in linking the establishment of sister chromatid cohesion with DNA replication in yeast [20]. We asked whether CUL4-DDB1, the putative functional homolog of Rtt101-Mms1 in human cells, participate in sister chromatid cohesion as well. To test this, we depleted CUL4A, CUL4B or DDB1 from 293T cells using small interfering RNA (siRNA) and measured sister chromatid cohesion. Cultured cells were harvested by trypsinization to enrich for cells in mitosis. Chromosome spreads were stained with Giemsa and the morphology of the mitotic cells was analyzed (Fig 1A). We did not synchronize cells in metaphase with nocodazole since vertebrate cohesins are removed from chromosome arms in prophase so that only centromere cohesion can be monitored [33]. We, however, wished to monitor cohesion not only at centromeres but also at telomeres and chromosome arms, where Rtt101-Mms1 have been shown to be required for cohesion establishment [20]. In our experiments, the term “normal cohesion” is defined as the state in which both centromere and chromosome arms are closely tethered each other (i, Fig 1A), whereas arm open (ii), partially separated but still paired (also called “railroad”, iii), unpaired (iv) or completely separated (v) chromatids indicate various extents of cohesion impairment. Under our experimental conditions, most chromatids in a single cell display similar morphology. We calculated the cohesion percentages as the proportion of “normal cohesion” cells (i) among total mitotic cells, where indicated. Alternatively, the percentage of cells bearing separated centromeres (iii, iv and v, Fig 1A) was used as an indicator as severe cohesion deficiency (S1 Fig). Depletion of either CUL4A or CUL4B reduced the normal cohesion from ~80% to ~40% (Fig 1B and 1C). The specificity of RNA interference (RNAi) was verified through complementation by over-expressing the respective proteins carrying a FLAG tag. This indicates that CUL4A and CUL4B may play at least partially non-redundant roles in sister chromatid cohesion. Similar results were observed for cells devoid of DDB1, whereas the cell cycle progression was not significantly affected (Fig 1D and S1A and S1B Fig). These results indicate that CUL4A, CUL4B and DDB1, like their homologs in yeast, are required for efficient cohesion in human cells.

Mms22 is one of the substrate adaptors of Rtt101-Mms1 in yeast. MMS22L, a putative human ortholog of Mms22, functions together with CUL4-DDB1 in replication-coupled nucleosome assembly [34]. Yet it remains to be proved whether it is a DCAF to date. To test this, we then co-expressed GFP-DDB1 and Flag-MMS22L in 293T cells. Flag-MMS22L was immunoprecipitated by anti-FLAG antibodies from whole cell extracts. As shown in Fig 1E, considerable amounts of DDB1 co-precipitated with Flag-MMS22L, arguing that MMS22L interacts with DDB1 and is likely a new DCAF of CRL4 ligases in human. Interestingly, MMS22L depletion resulted in significant cohesion defects at both chromosome arms and centromeres, reminiscent of the results from depletion of other CRL4 subunits CUL4A, CUL4B or DDB1 (Fig 1F and S1C Fig). Taken together, these data suggest that CRL4^MMS22L^ ligases participate in sister chromatid cohesion in human cells.

### CRL4^MMS22L^ ligases selectively interact with ESCO2

**Fig 2:**
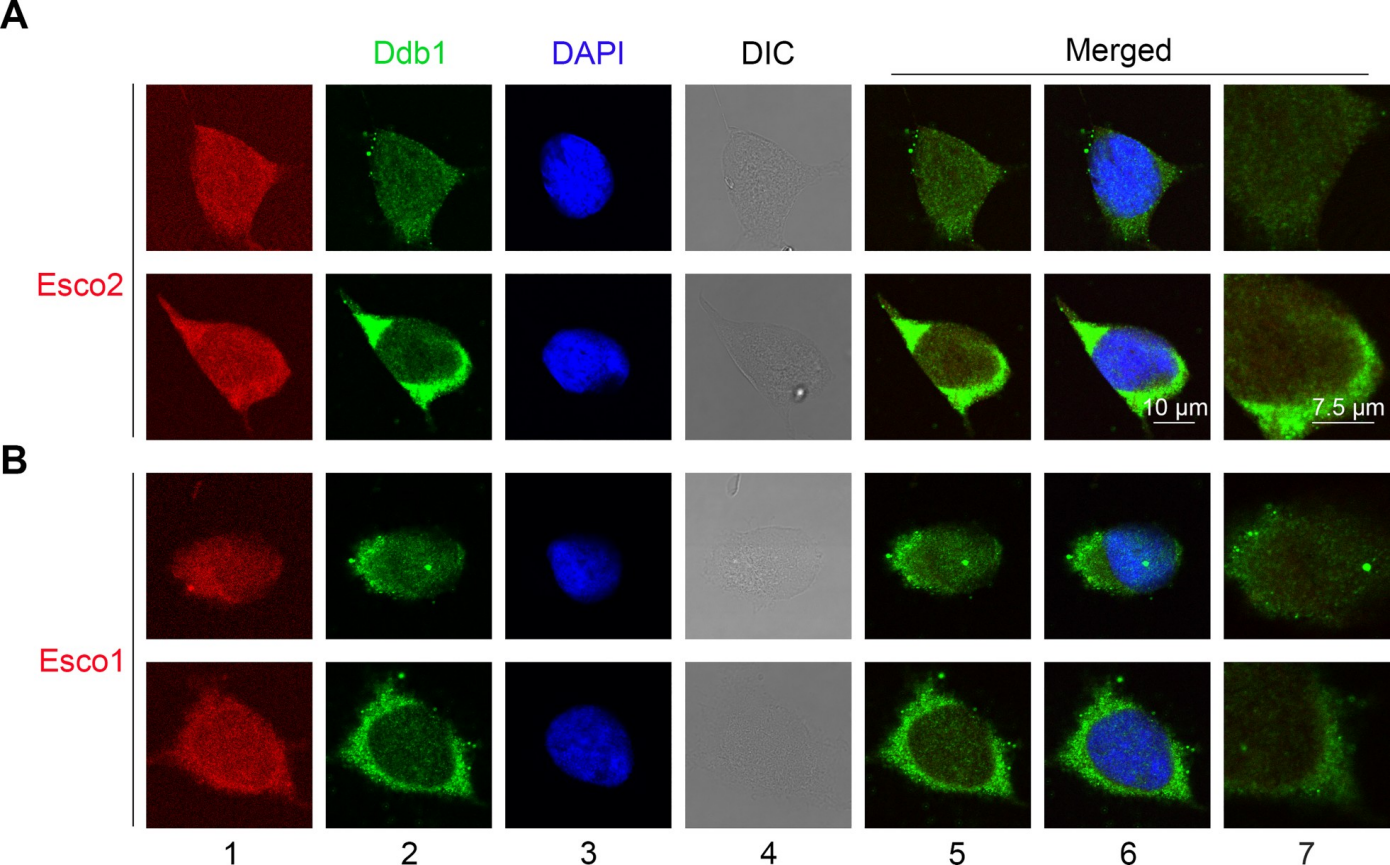
ESCO2 co-localizes with Cul4 and DDB1. (A, B) Immunofluorescence staining of endogenous DDB1, ESCO2 (A) or ESCO1 (B) proteins. Fixed cells were stained with the corresponding first antibodies and fluorescent second antibodies for confocal microscopy. Nuclei were stained with DAPI. The images were merged with (lane 6) or without DAPI (lane 5). See S2 Fig for the fluorescence images of ectopic expressed FP-tagged proteins.

To answer how CRL4s affect sister chromatid cohesion, we tested whether CRL4 subunits interact with the key cohesin acetyltransferases ESCO1 or ESCO2. We first examined the cellular distribution of ESCO1, ESCO2, CUL4A, CUL4B or DDB1 by immunofluorescence. RFP-labelled ESCO1 or ESCO2 and GFP-tagged CUL4A, CUL4B or DDB1 were introduced into 293T cells. In agreement with previous observations [23, 35], CUL4B localized to nucleus, whereas the others distributed throughout the whole cell (S2 Fig), as reported previously by other groups [29, 36]. We also detected the endogenous proteins by immunofluorescence staining with the corresponding antibodies. DDB1 seemed to be partially co-localize with ESCO2, but not with ESCO1 (Fig 2A and 2B).

**Fig 3:**
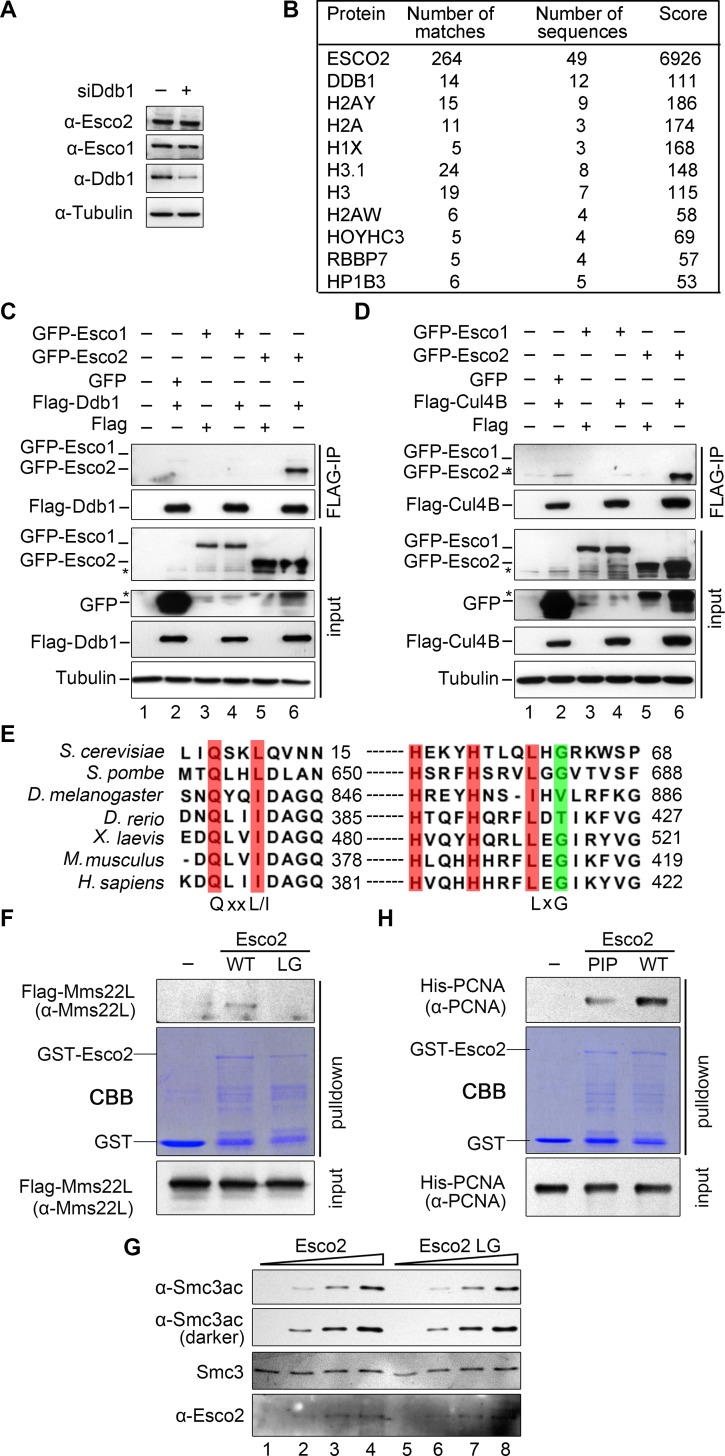
Preferential interaction between CRL4s^MMS22L^ and ESCO2. (A) The cellular levels of ESCO1 and ESCO2 proteins are not significantly changed upon DDB1-depletion. DDB1 RNAi was carried out as in Fig 1D. (B) DDB1 is repeatedly co-purified with ESCO2. 293T cells were co-transferred with *ESCO2-HF* plasmid. After tandem affinity purification, proteins in the final elution were analyzed by MS. Cells expressing ESCO1-HF were conducted as a control. (C, D) ESCO2, but not ESCO1, co-immunoprecipitates with CUL4A-CUL4B-DDB1-MMS22L. *GFP*, *GFP-ESCO1*, *GFP-ESCO2* and *Flag-DDB1* (C) or *Flag-CUL4B* (D) were co-expressed in 293T cells. FLAG-IP experiments were performed as described in Fig 1E. The asterisks indicate non-specific reacting bands. See S3 Fig for the data on *Flag-CUL4A* and *Flag-MMS22L* immunoprecipitation experiments. (E) The interaction motifs of Eco1/ESCO2 with PCNA and CRL4s are evolutionarily conserved from yeast to human. The amino acid sequences of Eco1/ESCO2 from the indicated organisms were aligned by CLC Genomics Workbench 3. See S3 Fig for alignment of yeast Eco1 and human ESCO1/2. (F) MMS22L interacts directly with ESCO2 through LxG motif. Recombinant GST-ESCO2 and its mutant proteins were expressed and purified for pull-down assays. FLAG-MMS22L was incubated with glutathione sepharose bound GST-ESCO2 or LG. Co-purified fractions were detected by western blotting. (G) ESCO2-LG mutant protein is potent in acetyltransferase activity in vitro. Purified ESCO2 or LG enzymes were subjected to in vitro acetyltransferase analysis using SMC3 as a substrate. Acetylated SMC3 was detected by SMC3ac-specific antibodies. (H) ESCO2 binds directly to PCNA through PIP box. His6-PCNA was subjected to GST pull-down assays as described in (F).

Given that CRL4s are ubiquitin ligases, we next compared the endogenous protein levels of ESCO1 and ESCO2 before or after DDB1-depletion. Both ESCO1 and ESCO2 were not significantly affected in the absence of DDB1, indicating that DDB1 ligases do not function through regulation of the global levels of cohesin acetyltransferases (Fig 3A). Meanwhile, in order to obtain insight into how ESCO2 is regulated, we searched for its interaction partners using affinity purification coupled mass spectrometry (AP-MS). To this end, ESCO2 carrying both His6 and 5FLAG tags was over-expressed in 293T cells and subjected to tandem affinity purification. Interestingly, DDB1, together with many histone subunits and chaperones (e.g., HP1), was repeatedly detected among the co-purified proteins with ESCO2-HF (Fig 3B).

We then performed immunoprecipitations to corroborate the interaction by ectopically expressing FLAG tagged subunit of CRL4s in 293T cells. Consistently, ESCO2 clearly co-precipitated with not only DDB1 (Fig 3C) but also other CRL4 subunits (Fig 3D and S3 Fig). On the contrary, virtually no ESCO1 was detectable in the precipitates of any CRL4 subunits in all experiments in parallel with ESCO2 (Fig 3C and 3D and S3 Fig). These data indicate that CRL4 ligases might have a preferential association with ESCO2.

### ESCO2 associates directly with MMS22L and PCNA

We had previously isolated a separation-of-function mutant in yeast, eco1-LG (L61DG63D), which shows a dramatically compromised interaction with Mms22 [20]. Interestingly, the two residues are highly conserved in ESCO2 (L415G417) (Fig 3E), but not in ESCO1 (S3C Fig), which correlates well with their different abilities to interact with CRL4s^MMS22L^. To validate whether these residues play a conserved role in the ESCO2 interaction, we performed a pull-down experiment. MMS22L was co-purified with GST-tagged ESCO2 WT, but barely with LG (L415DG417D) mutant protein (Fig 3F). On the other hand, LG mutant protein exhibited acetyltransferase activity similar to WT in vitro (Fig 3G), demonstrating that L415D and G417D mutations do not affect ESCO2’s catalysis. These results suggest a direct physical interaction between MMS22L and ESCO2, which is mediated by a conserved LxG motif in ESCO2. Meanwhile, using a similar approach, we showed that ESCO2 binds directly to PCNA, which is mainly mediated by the PIP box in ESCO2 (Fig 3E and 3H). It is noteworthy that both MMS22L and PCNA were hardly detectable in the ESCO2-IP/MS analysis mentioned in Fig 3B, implying that these interactions are likely weak or transient. Taken together, these data allow us to conclude that both MMS22L and PCNA are able to directly bind ESCO2 through evolutionarily conserved motifs LxG and PIP, respectively.

### ESCO2 functions in a CRL4^MMS22L^-dependent manner

**Fig 4:**
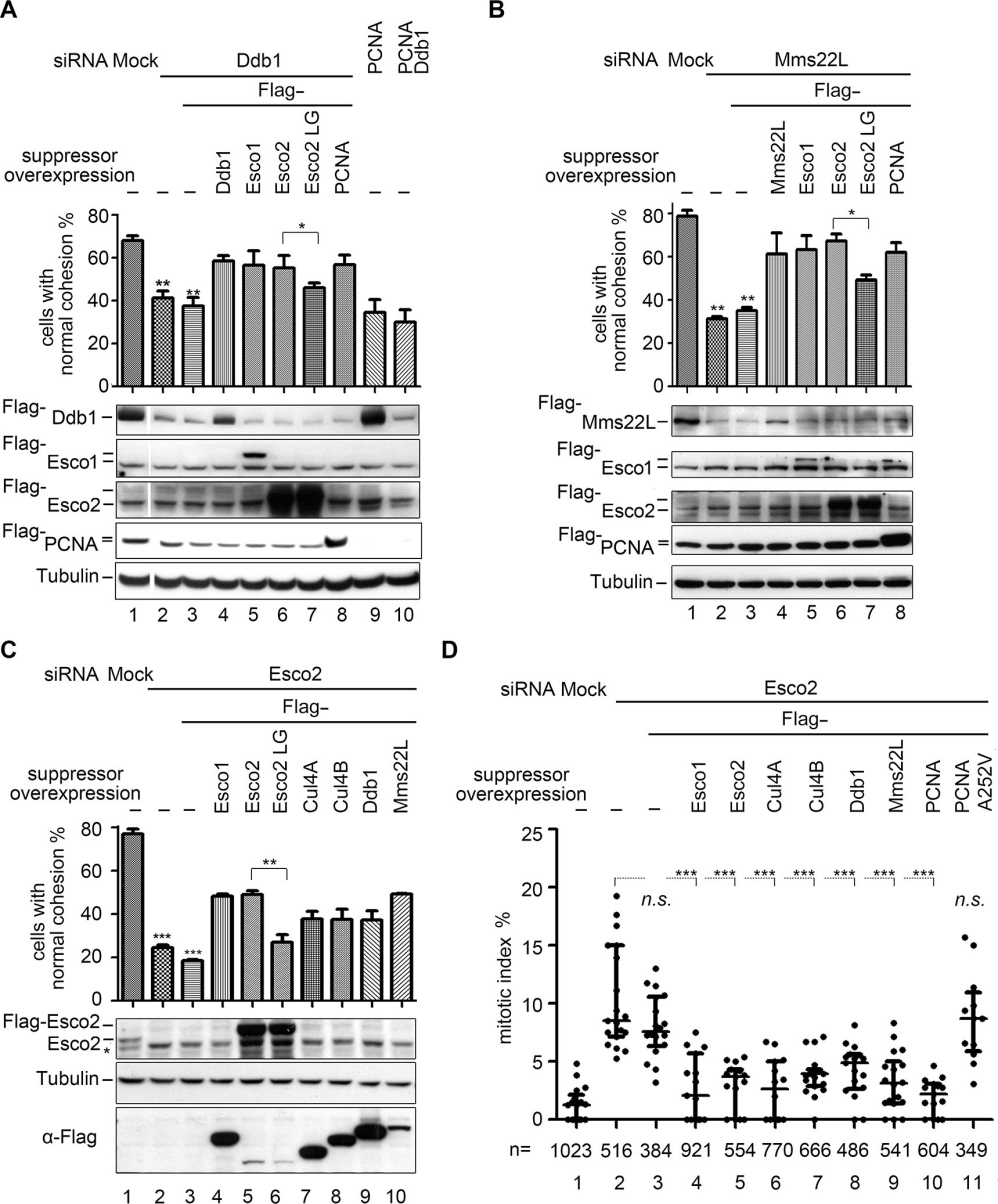
Functional interaction between CRL4s^MMS22L^ and ESCO2. (A) Over-expression of *ESCO1*, *ESCO2* or *PCNA* partially suppresses a cohesion defect in DDB1 knockdown cells, while over-expression of *ESCO2-LG* mutant does not. Immunoblots of DDB1, ESCO1, *ESCO2*, PCNA and Tubulin were shown below each column. The statistical significance was calculated via student’s *t*-test, ** P<0.01; * P<0.05. See S4A Fig for the corresponding *CEN* cohesion defect results. (B) *ESCO1*, *ESCO2* or *PCNA* over-expression partially compensates for a SCC defect caused by MMS22L knockdown, while over-expression of *ESCO2-LG* mutant does not. Immunoblots of MMS22L, ESCO1, ESCO2, PCNA and Tubulin are shown below each column. The statistical significance was calculated via student’s *t*-test, ** P<0.01; * P<0.05. See S4B Fig for the corresponding *CEN* cohesion defect results. (C) Over-expression of *CUL4A*, *CUL4B*, *DDB1*, or *MMS22L* partially suppresses the SCC defect caused by ESCO2 knockdown, while over-expression of an E3-interaction defective mutant, or ESCO2-LG (L415DG417D) mutant has no effect. ESCO2 was knocked down by siRNA in 293T cells, then Flag-tagged *ESCO1*, *ESCO2*, *ESCO2*-LG, *CUL4A*, *CUL4B*, *DDB1*, or *MMS22L* were overexpressed after siRNA delivery for 6 h. The percentage of cells in cohesion was determined as in Fig 1. Immunoblots of ESCO2, Flag and Tubulin from each RNAi experiment are shown below the corresponding column. The statistical significance was calculated via student’s *t*-test, *** P<0.001; ** P<0.01. See S4C Fig for the corresponding *CEN* cohesion defect results. See S4D Fig for the results of PCNA and PCNA-A252V alleles. (D) Over-expression of *CUL4A*, *CUL4B*, *DDB1*, *MMS22L* or *PCNA* suppresses the M phase arrest caused by ESCO2 knockdown. The portion of M-phase cells among total cells was counted after Hoechst 33342 staining. The statistical significance was calculated via student’s *t*-test, *** P<0.001, n: the total cell number counted.

Given the possible interaction between CRL4s and ESCO2, we asked whether lack of DDB1-MMS22L can be compensated by over-expressing ESCO2. To test this, we ectopically expressed *ESCO2* in a DDB1 (Fig 4A) or MMS22L depleted background (Fig 4B). Both mild and severe cohesion defects in either DDB1 or MMS22L-depleted 293T cells were markedly rescued by over-expression of *ESCO2* (Fig 4A and 4B, S4A and S4B Fig, lane 6), indicating a potent functional interaction between DDB1^MMS22L^ and ESCO2 as well as the physical interaction shown in Fig 3.

Over-expression of *ESCO2*-LG mutant led to a less suppression than that of wild-type (WT) *ESCO2* (Fig 4A and 4B, S4A and S4B Fig, compare lane 7 to 6), indicating that the role of ESCO2 is at least partially dependent on its interaction with DDB1^MMS22L^. In order to further address the contribution of the interaction between ESCO2 and CRL4s in sister chromatid cohesion, we tested the dosage suppression effects in an ESCO2-depleted background. In contrast to that of WT *ESCO2*, expression of the interaction-defective mutant *ESCO2*-LG hardly displayed suppression (Fig 4C and S4C Fig, compare lane 6 to 5). This result reinforces the argument that the interaction between ESCO2 and CRL4^MMS22L^ is important for the ESCO2’s role in cohesion establishment. Further supporting this, *CUL4*, *DDB1* and *MMS22L* were dosage suppressors of *ESCO2* knockdown mutant as well (Fig 4C and S4C Fig, lanes 7–10). These results implicate that DDB1^MMS22L^ might serve as a crucial positive regulator of the cohesion function of ESCO2.

Due to the fact that defects in sister chromatid cohesion often activate the spindle checkpoint and result in the G_2_/M arrest of the cell cycle, we then examined the proportion of M-phase cells (i.e., the mitotic index). The mitotic index was very low in untreated 293T cells, but rose to an average of ~12% when ESCO2 was depleted (Fig 4D, column 2), consistent with the observations from another group [37]. The G_2_/M arrest induced by ESCO2 knockdown was dramatically alleviated via over-expression of *CUL4A or CUL4B* (Fig 4D, columns 6 and 7), *DDB1* (column 8) or *MMS22L* (column 9). Taken together, these data demonstrate that the interaction between CRL4^MMS22L^ and ESCO2 is important for ESCO2 to function in sister chromatid cohesion and thereby mitotic progression.

Apart from interacting with CRL4s, the activity of Eco1 is also linked with replication forks through association with PCNA (Fig 3H) [19]. This notion was corroborated because the cohesion defects (S4D Fig) and mitotic arrest (Fig 4D, compare lanes 10 and 11) in ESCO2-depleted 293T cells were significantly rescued by over-expression of WT *PCNA*, but not by an ESCO interaction-defective mutant *PCNA*-A252V. Together, these data suggest that both CRL4^MMS22L^ and PCNA mediated interactions are critical for the ESCO2-dependent establishment of sister chromatid cohesion.

### CUL4-DDB1 ligases participate in sister chromatid cohesion by promoting ESCO2-mediated SMC3 acetylation

Given that the essential role of Eco1/ESCO lies in catalyzing SMC3 acetylation during cohesion establishment [13, 38], we next examined whether the dosage suppression effects observed above are due to facilitating SMC3 acetylation. For this purpose, SMC3 acetylation was measured in 293T cell lysates via immunoblots with an antibody that specifically recognizes SMC3K105ac/K106ac. S5A Fig demonstrates that the antibody recognizes an amount of SMC3ac proportional to the total protein concentrations. ESCO2-depleted cells displayed substantially reduced SMC3 acetylation (S5B Fig, compare lanes 1, 2 and 7), which was partially restored through ectopic expression of *DDB1* or *MMS22L* (S5B Fig, compare lanes 1, 5 and 6). Over-expression of either *CUL4A* or *CUL4B* stimulated SMC3 acetylation to a similar extent (S5B Fig, lanes 3 and 4). These results suggest that the compensation of cohesion defects in ESCO2-depleted cells by *CUL4*, *DDB1*, or *MMS22L* over-expression may be achieved through enhancing SMC3 acetylation.

**Fig 5:**
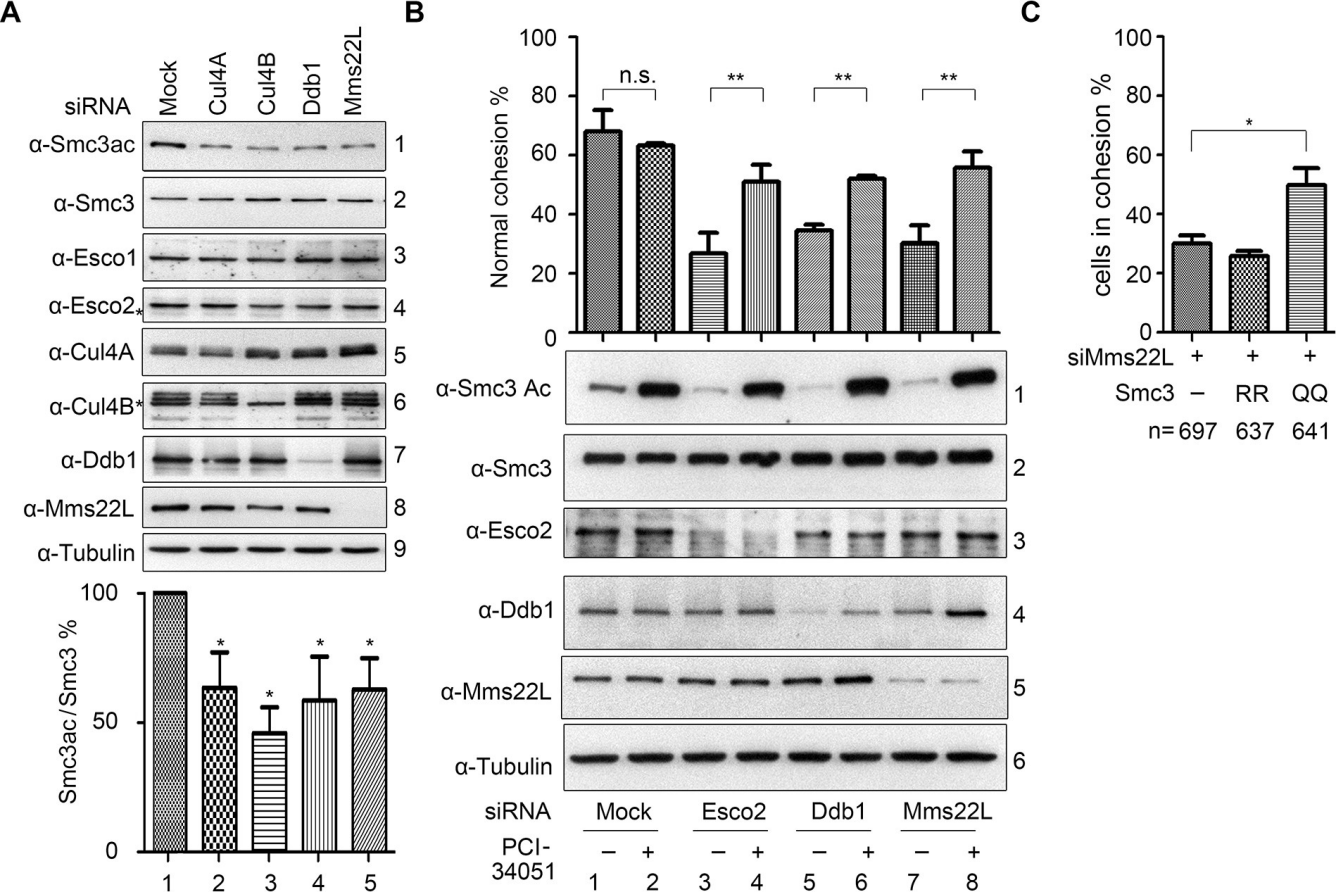
CUL4A-CUL4B-DDB1-MMS22L are required for ESCO2-dependent SMC3 acetylation. (A) Knockdown of CUL4-DDB1-MMS22L leads to reduced SMC3 acetylation. SMC3 acetylation was analyzed in the siRNA-transfected cells as above. The statistical significance from at least three independent repeats was calculated via student’s *t*-test, *P<0.05. See S5A Fig for the linear ranges of quantitation analysis of immunoblots, S5B Fig for the dosage suppression results of ESCO2-depleted cells, and S5C–S5E Fig for biological repeats. (B) Inhibition of deacetylase HDAC8 restores both SMC3ac and cohesion levels in DDB1/MMS22L –depleted cells. The indicated cells were cultured and treated with PCI-34051 for 3 h before collection and Giemsa analysis. Mitotic cells with normal cohesion were counted as described in Fig 1. The statistical significance was calculated via student’s *t*-test, ** P<0.01. (C) Overexpression of acetyl-mimic SMC3 mutant suppresses the cohesion defects caused by lack of MMS22L. MMS22L was knocked down by siRNA in 293T cells. 6 hr later, plasmids overexpressing SMC3RR (nonacetylated-mimic) or SMC3QQ (acetylation-mimic) were introduced. The cohesion percentage was determined as above.

This opens the possibility that CRL4s directly participate in regulating SMC3 acetylation. Depletion of each subunit of CRL4s led to moderately compromised SMC3 acetylation (Fig 5A and S5C–S5E Fig), indicating that CRL4s^MMS22L^ are required for efficient ESCO2-dependent SMC3 acetylation. Meanwhile, the protein levels of both ESCO enzymes were not significantly affected (Fig 5A, descending panels 3 and 4), suggesting that CUL4-DDB1-MMS22L unlikely regulate the expression and/or protein turnover of ESCO1 and ESCO2.

To further validate the role of CRL4s in SMC3 acetylation, we then set out to determine whether inhibition of HDAC8 is able to restore compromised SMC3 acetylation and cohesion caused by CRL4^MMS22L^ -depletion. Since HDAC8 is the deacetylase of SMC3 [39], we treated proliferating cells with the HDAC8 inhibitor, PCI-34051. Both SMC3 acetylation and cohesion efficiency increased markedly in WT and Esco2-depleted cells in the presence of PCI-34051 (Fig 5B, lanes 1–4), as reported previously [32]. The drug had a similar increase in the levels of SMC3ac and normal cohesion when DDB1 and MMS22L were depleted individually (lanes 5–8). Nevertheless, Shirahige’s group shows that PCI-34051 is not able to restore the SMC3ac levels caused by compromised PDS5A-PDS5B-ESCO1 branch [32]. This supports that DDB1 and MMS22L function in the ESCO2-catalyzed SMC3 acetylation pathway, which can be reversed by HDAC8. Consistently, overexpression of the acetylation-mimic SMC3QQ mutant rescues the cohesion defects caused by MMS22L-depletion (Fig 5C). Taken together, these data reinforce the notion that CRL4^MMS22L^ ligases modulate the activity of ESCO2 on SMC3 acetylation, and thus cohesion establishment.

### Both CRL4s and PCNA help to stabilize ESCO2 on chromatin

**Fig 6:**
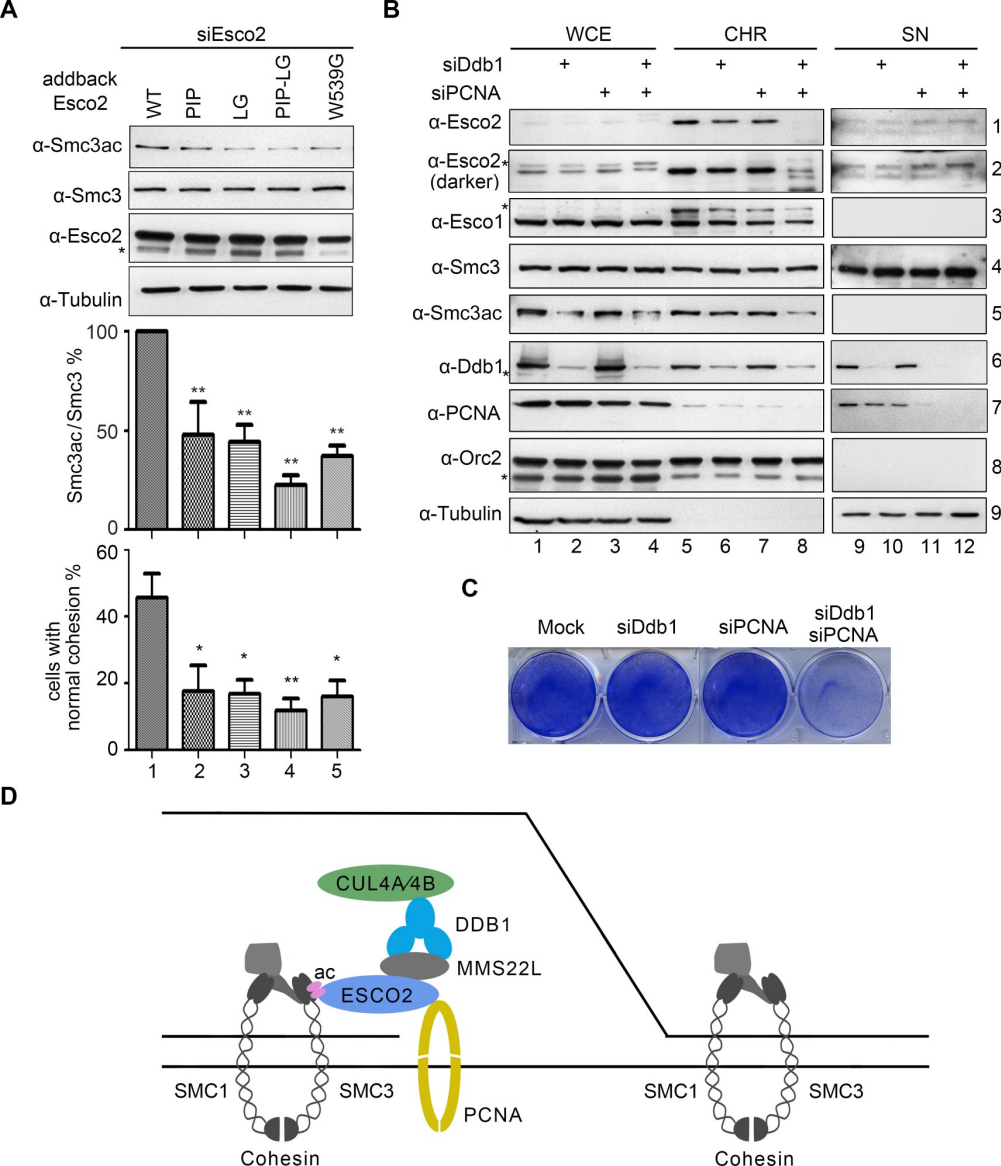
Both CRL4s and PCNA mediated interactions are required for stabilizing ESCO2 on chromatin. (A) *ESCO2* mutants display compromised SMC3 acetylation and thereby cohesion establishment. *ESCO2* alleles: PIP, *ESCO2*-Q374AI376AI377A; LG, *ESCO2*-L415DG417D; PIP-LG, *ESCO2*-Q374AI376AI377A-L415DG417D. Quantitation from three biological repeats is shown for SMC3ac (middle) and cohesion efficiency (lower). The statistical significance was calculated via student’s *t*-test, *P<0.05, **P<0.01. (B) DDB1 and PCNA are required for the chromatin association of ESCO2. Chromatin fractions were prepared from cells transfected with the DDB1 or PCNA siRNAs. Orc2 served as a loading control of the chromatin-enriched fraction (CHR). (C) Combined depletion of DDB1 and PCNA caused cell death. Cells were stained with Coomassie brilliant blue 48 h after transfection with the indicated siRNAs. (D) A co-regulation model of ESCO2 by CRL4s and PCNA in human cells. Whilst replication fork proceeding during S phase, ESCO2 is recruited via interactions with fork components including PCNA and CRL4^MMS22L^ ligases. This is required for efficient SMC3 acetylation of the pre-loaded cohesin ring, which triggers the establishment of cohesion between two newly synthesized sister chromatids.

Next, we directly tested whether the regulation of CRL4s on the ESCO2 activity depends on their physical interactions. For this purpose, we examined the phenotypes of several *ESCO2* alleles defective in either CRL4s-binding (*ESCO2*-LG) or PCNA-binding (*ESCO2*-PIP) shown in Fig 3. To obtain a catalytic-deficient enzyme, we also introduced the missense mutation W539G in ESCO2, which occurs frequently in Roberts Syndrome (RBS) patients [40]. Indeed, the W539G allele showed a substantial decrease in SMC3 acetylation (Fig 6A, lane 5), in agreement with its location within the acetyltransferase domain. In addition, *ESCO2*-LG exhibited a significant decrease in SMC3 acetylation and cohesion efficacy to a similar extent as the catalytic-deficient W539 allele (Fig 6A, lane 3), indicating that CRL4s-mediated interaction is crucial for ESCO2 operating on SMC3. Similarly, a PCNA-interaction defective allele, *ESCO2*-PIP, reduced SMC3 acetylation and cohesion efficacy as well (lane 2). Interestingly, when we combined both mutations on ESCO2 (ESCO2-LG-PIP), we found a synergistic decline in SMC3 acetylation and cohesion (Fig 6A, lane 4). These data suggest that the function of ESCO2 is cooperatively regulated through its dual interactions with both CRL4^MMS22L^ and PCNA.

Since both CRL4^MMS22L^ and PCNA associate with replication forks, we then analyzed the contribution of DDB1 and PCNA to recruiting/stabilizing ESCO2 on chromatin. Chromatin fractions were prepared from the 293T cells transfected with siRNAs specific to DDB1 or PCNA. Depletion of either DDB1 or PCNA led to moderately reduced amounts of ESCO2 on chromatin (Fig 6B, lanes 6 and 7), whilst the combinational depletion of DDB1 and PCNA only produced a subtle effect on the total ESCO2 levels (Fig 6B, WCE, lane 4). However, a clear synergistic loss of ESCO2 on chromatin was observed (CHR, lane 8). Consistently, the level of acetylated SMC3 largely reduced whereas the total SMC3 protein on chromatin remained virtually unaffected (Fig 6B, descending panels 4 and 5). Meanwhile, the chromatin-associated ESCO1 level only displayed a mild change (Fig 6B, lane 8, panel 3). In good agreement with this, only when DDB1 and PCNA depletions were combined, dramatic cell death was observed by live cell staining (Fig 6C). These data suggest a cooperative mechanism for CRL4s and PCNA to properly target the essential cohesin acetyltransferase ESCO2 on its substrate SMC3, which contributes to the coupling between the establishment of sister chromatid cohesion and replication fork progression in human cells (Fig 6D).

## Discussion

How sister chromatid cohesion is established in mammals remains largely unclear. Here we have identified an evolutionarily conserved mechanism of CRL4 ubiquitin ligases, together with PCNA, in regulation of DNA replication-coupled cohesion establishment in human cells.

The essential step to establish cohesion is SMC3 acetylation by Eco1 in yeast or ESCO in human [13–15]. Precise control of the reaction is required for this essential cellular process. One of the main findings of this study is that human CRL4^MMS22L^ ligases exclusively interact with and preferentially regulate ESCO2. Despite the fact that both ESCO1 and ESCO2 catalyze acetylation of SMC3, their temporal regulation is distinct from each other [32, 37, 41]. ESCO1 acetylates SMC3 in a Pds5-dependent manner before and after DNA replication [37], whereas ESCO2 is believed to function during S phase. Our findings provide molecular details of how ESCO2 is controlled in a DNA replication-coupled fashion through dual interaction with CRL4^MMS22L^ and PCNA in human cells.

During the revision of this manuscript, Peters’s group reported that ESCO2 is recruited to chromatin via direct association with MCMs, the core of eukaryotic replicative helicase Cdc45-Mcm2-7-GINS [42]. Moreover, Zheng et al found that MCMs also associate with cohesin and its loader, which promotes cohesin loading during S phase [43]. It’s worth noting that the contributions of MCM, PCNA and CRL4s –mediated interactions to ESCO2 regulation are not mutually exclusive, because the defects in one of these interactions only cause partial loss of the essential function of ESCO2. A very interesting finding in their work is that ESCO2 binds MCMs predominantly in the context of chromatin in spite of the fact that there are considerably excessive amounts of MCMs in nucleoplasm [42]. Even among the abundant chromatin-loaded MCM rings, only a small portion are activated and assembled into replication forks [44, 45]. How ESCO2 is specifically recognized by the activated MCMs and travels with replication fork has therefore not been addressed yet. But, the interactions of ESCO2 with PCNA and CRL4^MMS22L^ identified previously [19] and in this study, albeit relatively weak or transient, may contribute to the preferential association of ESCO2 with the activated MCMs on replication fork. Intriguingly, in HeLa cells, MMS22L-TONSL bind MCMs as well as replication-coupled H3.1-H4 [46–51]. Therefore, it will be of great interest to test the functional interplay among these fork-associated factors in the future.

In addition to these interactions, CRL4s have been found involved in multiple replication-coupled chromatid events. For instance, CUL4-DDB1 (Rtt101-Mms1) ubiquitylates histone H3-H4, which elicits the new histone hand-off from Asf1 to other chaperones for chromatin reassembly in both yeast and human cells [34]. Another CRL4, CRL4^WDR23^, ubiquitylates SLBP to activate histone mRNA processing and expression during DNA replication [52]. Further studies are needed to illustrate the details of crosstalk among these replication-coupled events, for instance, nucleosome assembly and cohesion establishment in human cells.

Over-expression of CUL4, DDB1 and MMS22L has been reported to correlate with lung and esophageal carcinogenesis [53], implicating them as key genome caretakers. Moreover, mutations in *ESCO2* gene cause Roberts Syndromes with a predisposition to cancer [40]. The functional interplay between CUL4-DDB1 and ESCO2 identified here will shed new light on understanding the etiology of these human diseases.

## Materials and methods

### Cell culture and RNAi

HEK293T cells were cultured in DMEM media supplemented with 10% fetal bovine serum (FBS, Gibco) at 37°C with 5% CO_2_. For RNAi experiments, cells were transfected with 80 nM siRNAs using Lipofectamine 3000 (Invitrogen) for 48 h following the manufacturer’s instructions. Over-expression plasmid or control plasmid for target genes was transferred into the cells when necessary. For HDAC8 inhibition experiments, 6.25 μM PCI-34051 (Selleckchem) was applied 3 h before harvest. Immunoblotting with specific antibodies was used to confirm the downregulation of the targets. The sequences of siRNA oligos used in this study are listed in Table 1. All siRNA oligos were synthesized by Sangon Biotech, China.

**Table 1:** The sequences of siRNA oligos.

Target gene	sequences of siRNA oligos
Esco1	5’-CCAGUGUUGAAAGACAAAUACUUCA-3’
	5’-GGACAAAGCUACAUGAUAG-3’
Esco2	5’-GACCCAACACCAGAUGGCAAGUUAU-3’
	5’-ACAGAAGAGUUUAACUGCUAAGUAU-3’
Cul4A	5’-GAAGCUGGUCAUCAAGAAC-3’
	5’-GACAAUCCGAAUCAGUACC-3’
Cul4B	5’-AAGCCUAAAUUACCAGAAA-3’
	5’-AGAUAAGGUUGACCAUAUA-3’
Ddb1	5’-CGUUGACAGUAAUGAACAAGGCUCC-3’
	5’-CCUGUUGAUUGCCAAAAAC-3’
Mms22L	5’-UCACAAAGUCCUUGGAAUA-3’
	5’-AAGACUUGCUGUUGCGAUA-3’
PCNA	5’-GGAGGAAGCUGUUACCAUA-3’
	5’-CGGUGACACUCAGUAUGUC-3’
Mock	5’-UUCUCCGAACGUGUCACGU-3’

### Plasmid construction

Trizol reagent (CWBIO) was used to isolate total RNA according to the manufacturer’s instructions. cDNA was synthesized using reverse transcriptase (Promega). Full length genes studied were inserted to the prk5-Flag, GFP or mCherry vectors [54]. The prk5-Flag vector was kindly provided by Dr. Jun Tang (China Agricultural University) and was replaced by GFP or mCherry when necessary.

### Recombinant protein purification, pull-down and in vitro acetyltransferase assays

Recombinant GST-tagged ESCO2 or its derivatives were expressed and purified according to the GE healthcare’s instructions. Pull-down and in vitro acetyltransferase assays were performed as described previously [20].

### Chromosome spreads

Chromosome spreads were performed as described in [43], with minor modifications. In brief, cultured cells were harvested by trypsinization and then 75 mM KCl was used as hypotonic treatment. Cells were fixed with methanol and acetic acid (3:1) three times and then dropped onto the slides. After half an hour, cells were stained with 0.05% Giemsa (Merck) for 10 min at room temperature. Images were captured using a Leica microscope equipped with a 100×/NA1.3 oil objective. The incidence of sister chromatid separation was determined from at least 200 mitotic cells and all experiments were repeated at least three times. In our experimental conditions, almost all chromosomes within a single cell display similar cohesion defects. Total cohesion defects were defined as cells exhibiting precocious separation of both arms and centromeres, while *CEN* cohesion defects shown in the Supporting Figures were calculated as the percentages of cells bearing separated centromeres among mitotic cells.

### Cell extract, immunoprecipitation and immunoblotting

Cells were washed twice with PBS. To obtain whole cell extracts for immunoblotting, cells were resuspended with RIPA buffer (50 mM Tris-HCl, 250 mM NaCl, 1% TritonX-100, 0.25% Sodium deoxycholate, 0.05% SDS, 1 mM DTT) and lysed on ice for 20 min. For immunoprecipitation experiments, cells were resuspended in lysis buffer (50 mM Tris-HCl, 150 mM NaCl, 1% NP-40, 5 mM EDTA, 10% glycerin) and incubated on ice for 20 min, then sonicated for 30 sec. For each sample, 250 μg total protein was incubated with anti-Flag agarose for 1.5 h at 4°C, then washed five times with lysis buffer. All samples were run on sodium dodecyl sulfate polyacrylamide gel electrophoresis (SDS-PAGE) and transferred to PVDF membranes. Signals were detected with specific antibodies using eECL Western Blot Kit (CWBIO).

### Affinity purification coupled to mass spectrometry (AP-MS)

ESCO2-HF was purified from whole cell extracts by anti-FLAG M2 (Sigma) and Ni^2+^ affinity gels successively. Nonspecific bound proteins were removed by washing with 0.25 μg/μl FLAG peptide. Bound fraction was eluted by 2 μg/μl FLAG peptide and 300 mM imidazole, respectively. An untagged cell line and ESCO1-HF were subjected to the same procedure as controls. The final eluates were analyzed by mass spectrometry analysis (Q Exactive Hybrid Quadrupole-Orbitrap Mass Spectrometer, Thermo Fisher). The procedures were repeated three times for both ESCO2-HF and ESCO1-HF to identify the different interactors of ESCO2 and ESCO1.

### Immunofluorescence or fluorescence microscopy

Cells were grown in cover glasses placed into Nunc 6-well plates. After being fixed with 4% paraformaldehyde, cells were blocked in PBS containing 2% BSA for 30 min prior to incubation with desired antibodies at 4°C overnight. After three washes with PBST, fluorescent secondary antibodies were added for 1 hr at room temperature. Cells were again washed three times with PBST and stained with 1 μg/ml DAPI for 5 min. Images were captured with a laser‑confocal microscope (DMi8; Leica Microsystems).

For overexpressing FP-tagged proteins, cells were transfected with plasmids using Lipofectamine 3000 (Invitrogen) for 24 h according to the instructions. The slides were viewed as above.

### Antibodies

Antibodies used in this work were as below: ESCO1 (Abcam, ab180100), ESCO2 (Abcam, ab86003), CUL4A (proteintech, 14851-1-AP), CUL4B (Proteintech, 12916-1-AP), DDB1 (Abcam, ab9194), MMS22L (Abcam, ab181047), SMC3 (BETHYL, A300-060A), acetylated SMC3 (Merck, MABE1073), Orc2 (CST, #4736), Tubulin (MBL, PM054) and PCNA (Santa Cruz, sc-56).

## Supporting information

S1 Fig (Related to Fig 1). (A) Depletion of DDB1 causes neglectable changes in cell cycle progression. Representative cell cycle profiles of 293T cells after release from double thymidine block were monitored by flow cytometry.
(B, C) The percentages of 293T cells bearing cohesion defects in centromeres (groups iii, iv and v in Fig 1A) were calculated for each sample as shown in Fig 1D and 1F. The statistical significance was calculated via student’s t-test, *** P<0.001; ** P<0.01.
(TIF)

S2 Fig (Related to Fig 2). (A) Localization of ESCO2, CUL4A, CUL4B and DDB1. 293T cells were co-transferred with RFP-ESCO2 plasmids and GFP, GFP-CUL4A, GFP-CUL4B, or GFP-DDB1 plasmids. After 24 h, nuclei were stained with DAPI. Pictures were captured with a laser-confocal microscope. RFP and GFP images were merged with (lane 6) or without DAPI (lane 5).
(B) ESCO1 does not co-localize with CRL4s. 293T cells were co-transferred with RFP-ESCO1 plasmids and GFP, GFP-CUL4A, GFP-CUL4B, or GFP-DDB1 plasmids. Fluorescence microscopy were conducted as described above.
(TIF)

S3 Fig (Related to Fig 3). (A, B) ESCO2, but not ESCO1, co-immunoprecipitates with CUL4A-CUL4B-DDB1-MMS22L. GFP, GFP-ESCO1, GFP-ESCO2 and Flag- CUL4A (A) or Flag-MMS22L (B) were co-expressed in 293T cells. FLAG-IP experiments were performed as described in Fig 3B. The asterisks indicate non-specific reacting bands.
(C) The LG motif (L415G417, labelled with asterisks) required for interaction with CRL4^MMS22L^ exists in yeast Eco1, human ESCO2, but not in human ESCO1. The alignment of protein sequence was conducted via CLC Genomics Workbench 3. The secondary structures were adapted from the crystal structure of hESCO1 (PDB: 5n22).
(TIF)

S4 Fig (Related to Fig 4). (A) The percentages of cells bearing cohesion defects at centromeres were calculated as described in S1 Fig. The statistical significance was calculated via student’s t-test, *** P<0.001; ** P<0.01; * P<0.05. See also Fig 4A.
(B) The percentages of cells bearing cohesion defects at centromeres were calculated as described in S1 Fig. The statistical significance was calculated via student’s t-test, ** P<0.01; * P<0.05. See also Fig 4B.
(C) The percentages of cells bearing cohesion defects at centromeres were calculated as described in S1 Fig. The statistical significance was calculated via student’s t-test, ** P<0.01. See also Fig 4C.
(D) PCNA WT, not an interaction-defective allele PCNA-A252V, is a dosage suppressor of ESCO2-depletion mutant. See also Fig 4D.
(TIF)

S5 Fig (Related to Fig 5). **CRL4MMS22L are required for efficient SMC3 acetylation**.
(A) Quantitation of protein levels via western blotting. Immunoblots of SMC3, SMC3ac and tubulin using the corresponding antibodies. Titrations of 293T cell extracts (10–80 μg total proteins) were applied for western blot. Quantitation of acetylated SMC3, SMC3 and tubulin proteins among total input proteins. The intensity of each band was quantified by Quantity One (Bio-Rad) and plotted to validate that the protein levels are proportional to the total inputs within the range tested.
(B) Over-expression of CRL4 subunits is able to partially restore the levels of Smc3ac caused by ESCO2 depletion. The representative immunoblots (upper) along with the relative SMC3ac levels of three experiments (lower) are shown. SMC3ac stands for acetylated SMC3. The statistical significance was calculated via student’s t-test.
(C-E) Representative biological repeats of Fig 5A.
(TIF)
