# Supplementary figures and images for: Cul4-Ddb1 ubiquitin ligases facilitate DNA replication-coupled sister chromatid cohesion through regulation of cohesin acetyltransferase Esco2
Source: PLoS Genet. 2019 Feb 19;15(2):e1007685. doi: 10.1371/journal.pgen.1007685 (PMC6396947; doi:10.1371/journal.pgen.1007685)

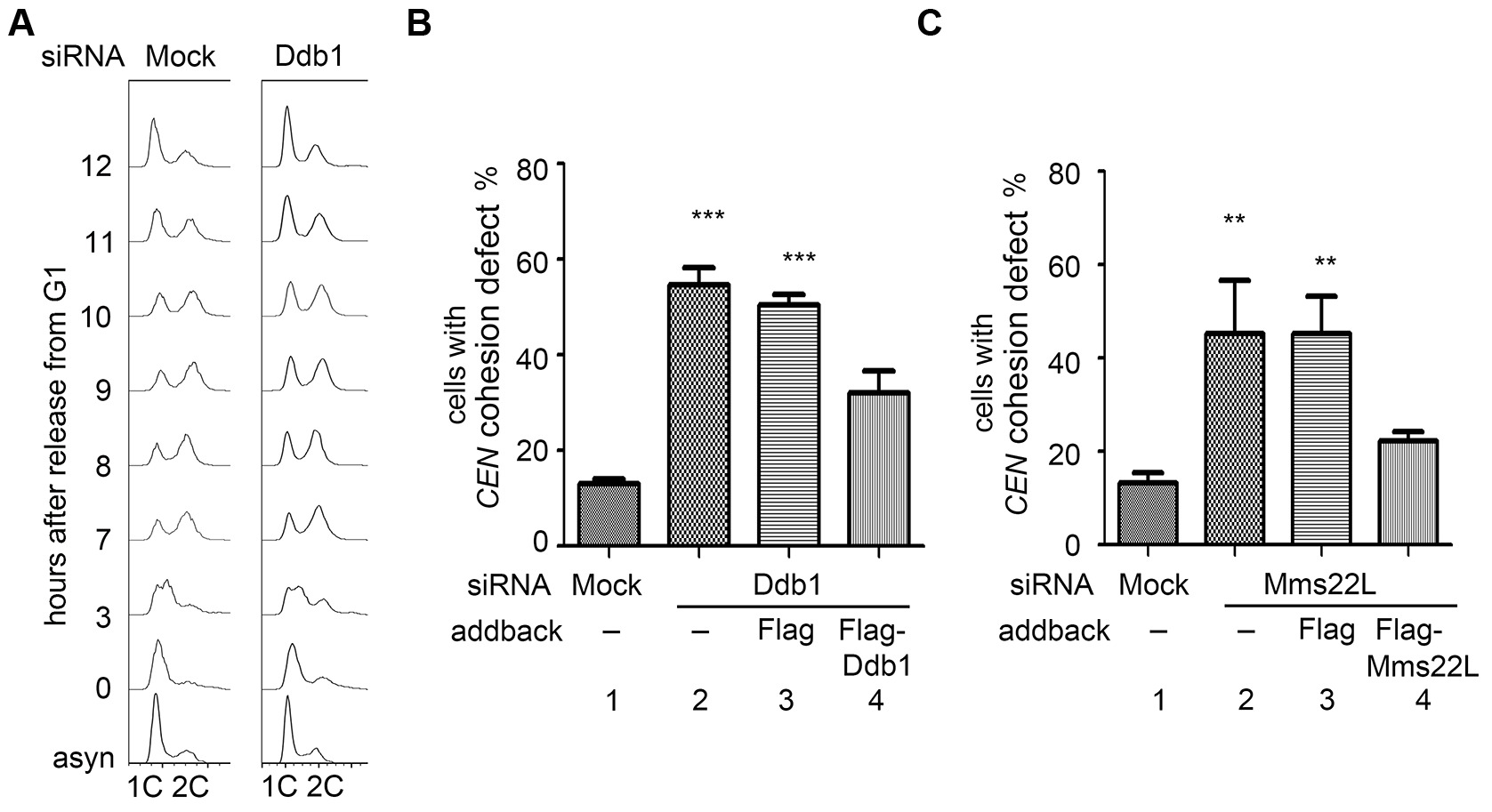

Supplement: S1 Fig — (A) Depletion of DDB1 causes neglectable changes in cell cycle progression. Representative cell cycle profiles of 293T cells after release from double thymidine block were monitored by flow cytometry. (B, C) The percentages of 293T cells bearing cohesion defects in centromeres (groups iii, iv and v in Fig 1A) were calculated for each sample as shown in Fig 1D and 1F. The statistical significance was calculated via student’s t-test, *** P<0.001; ** P<0.01. (TIF) [file pgen.1007685.s001.tif]

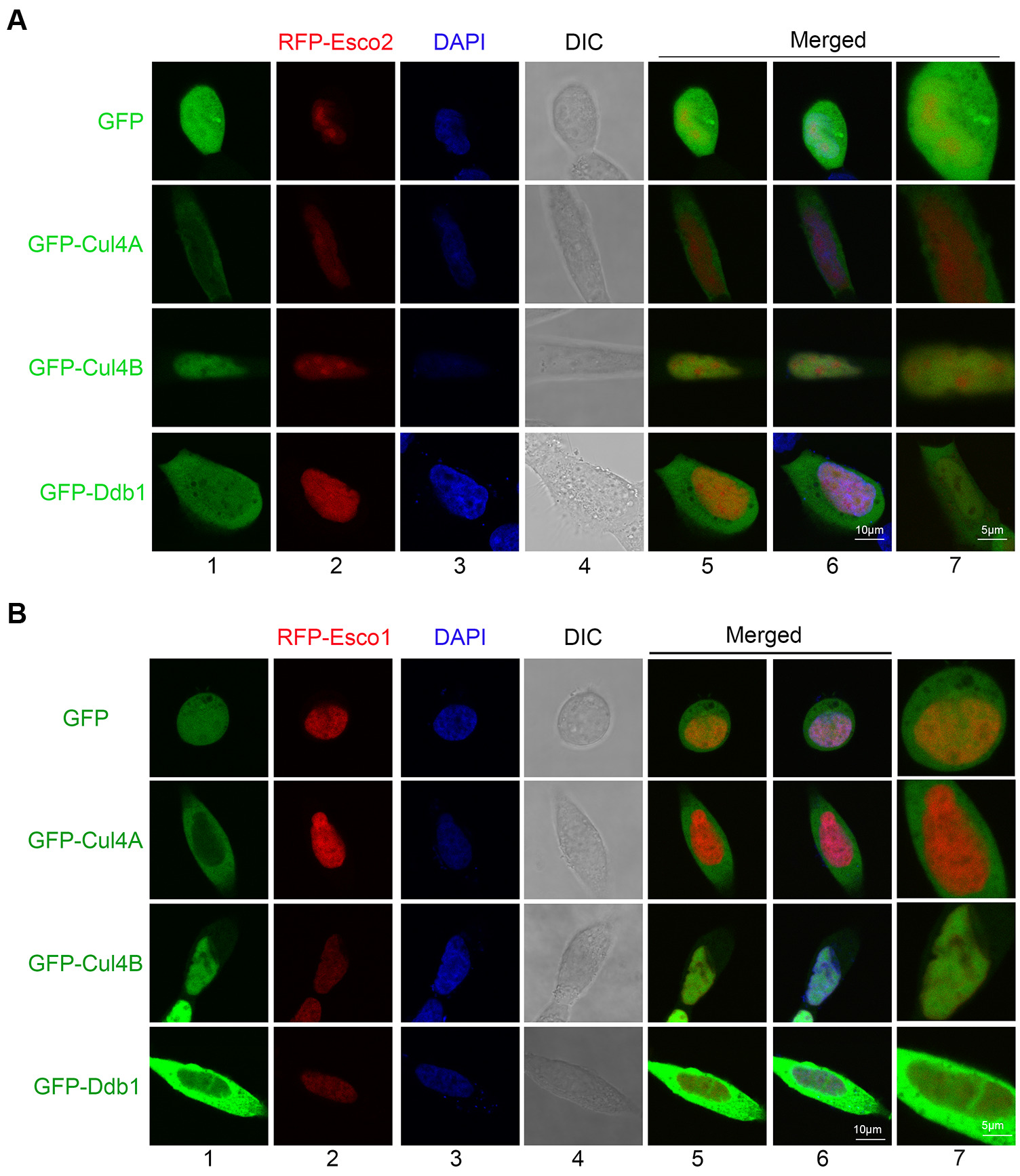

Supplement: S2 Fig — (A) Localization of ESCO2, CUL4A, CUL4B and DDB1. 293T cells were co-transferred with RFP-ESCO2 plasmids and GFP, GFP-CUL4A, GFP-CUL4B, or GFP-DDB1 plasmids. After 24 h, nuclei were stained with DAPI. Pictures were captured with a laser-confocal microscope. RFP and GFP images were merged with (lane 6) or without DAPI (lane 5). (B) ESCO1 does not co-localize with CRL4s. 293T cells were co-transferred with RFP-ESCO1 plasmids and GFP, GFP-CUL4A, GFP-CUL4B, or GFP-DDB1 plasmids. Fluorescence microscopy were conducted as described above. (TIF) [file pgen.1007685.s002.tif]

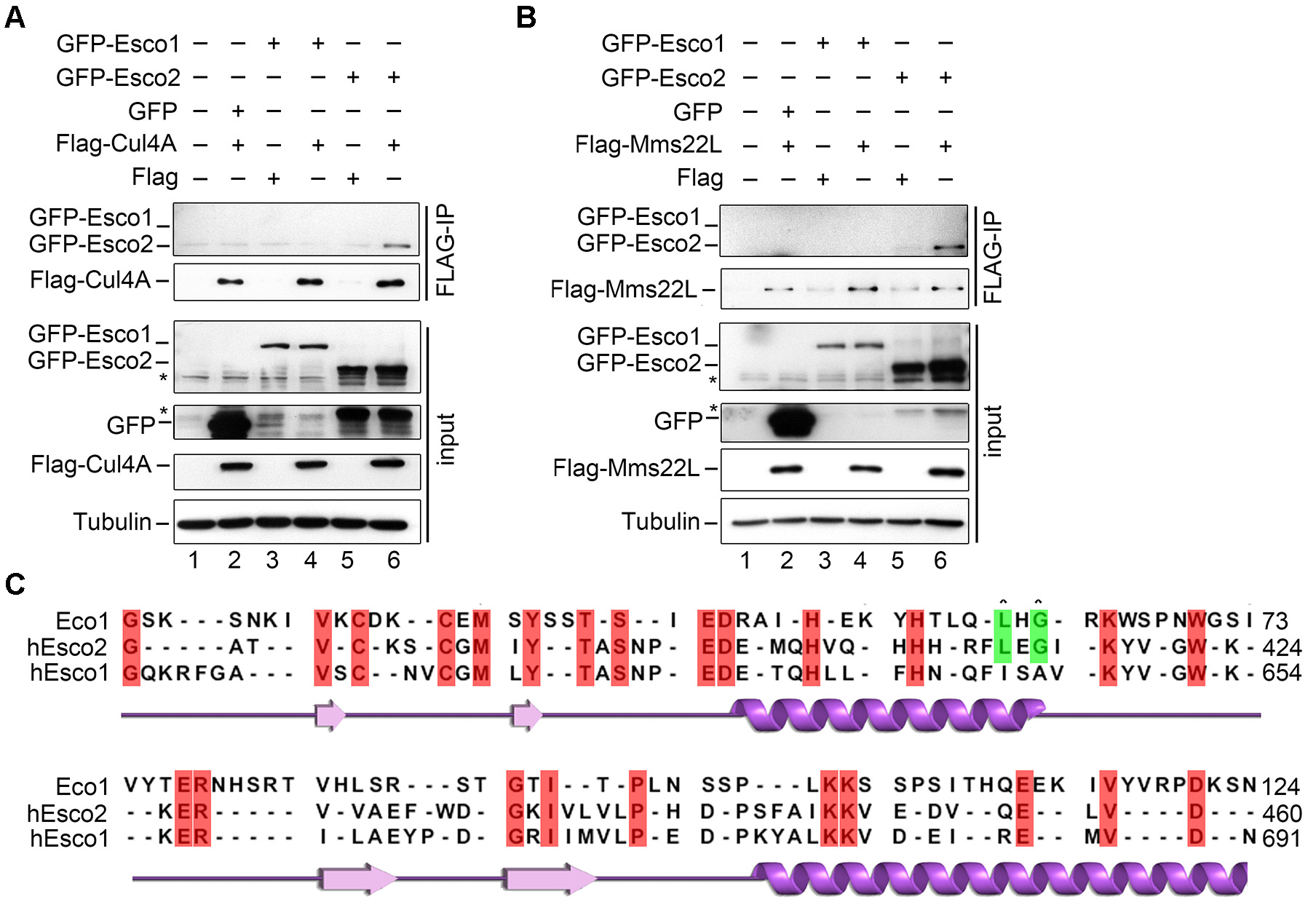

Supplement: S3 Fig — (A, B) ESCO2, but not ESCO1, co-immunoprecipitates with CUL4A-CUL4B-DDB1-MMS22L. GFP, GFP-ESCO1, GFP-ESCO2 and Flag- CUL4A (A) or Flag-MMS22L (B) were co-expressed in 293T cells. FLAG-IP experiments were performed as described in Fig 3B. The asterisks indicate non-specific reacting bands. (C) The LG motif (L415G417, labelled with asterisks) required for interaction with CRL4MMS22L exists in yeast Eco1, human ESCO2, but not in human ESCO1. The alignment of protein sequence was conducted via CLC Genomics Workbench 3. The secondary structures were adapted from the crystal structure of hESCO1 (PDB: 5n22). (TIF) [file pgen.1007685.s003.tif]

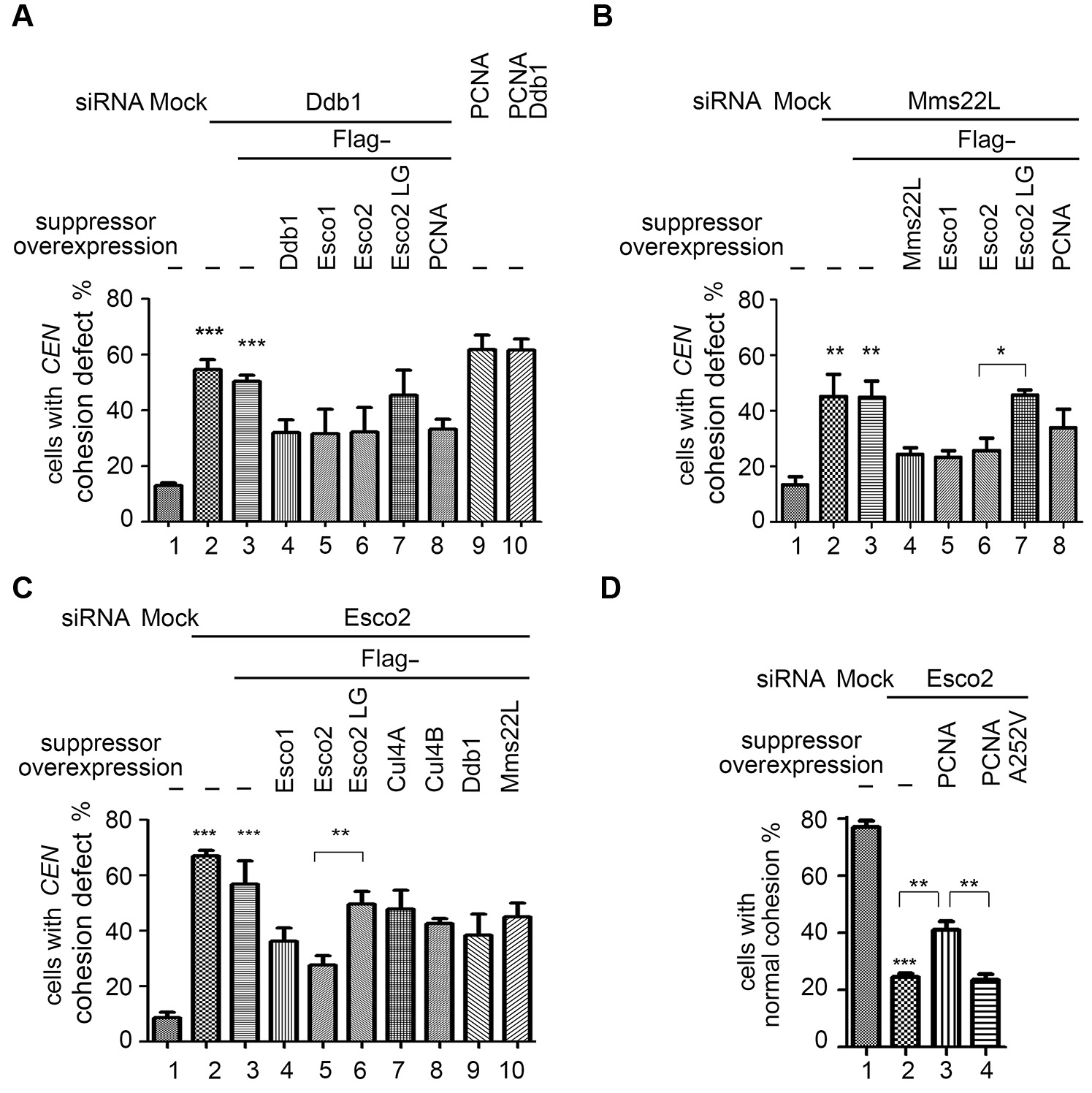

Supplement: S4 Fig — (A) The percentages of cells bearing cohesion defects at centromeres were calculated as described in S1 Fig. The statistical significance was calculated via student’s t-test, *** P<0.001; ** P<0.01; * P<0.05. See also Fig 4A. (B) The percentages of cells bearing cohesion defects at centromeres were calculated as described in S1 Fig. The statistical significance was calculated via student’s t-test, ** P<0.01; * P<0.05. See also Fig 4B. (C) The percentages of cells bearing cohesion defects at centromeres were calculated as described in S1 Fig. The statistical significance was calculated via student’s t-test, ** P<0.01. See also Fig 4C. (D) PCNA WT, not an interaction-defective allele PCNA-A252V, is a dosage suppressor of ESCO2-depletion mutant. See also Fig 4D. (TIF) [file pgen.1007685.s004.tif]

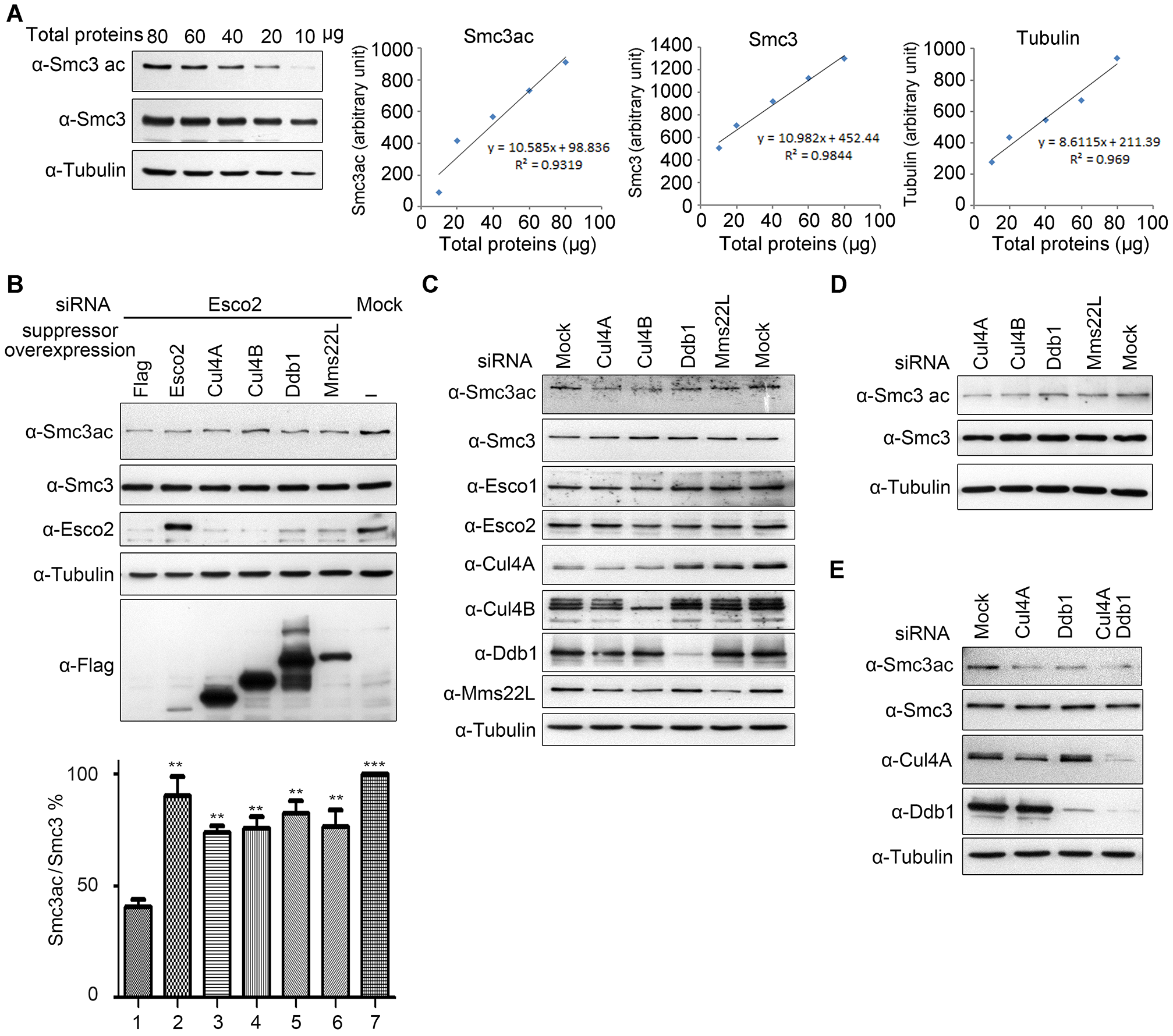

Supplement: S5 Fig — CRL4MMS22L are required for efficient SMC3 acetylation. (A) Quantitation of protein levels via western blotting. Immunoblots of SMC3, SMC3ac and tubulin using the corresponding antibodies. Titrations of 293T cell extracts (10–80 μg total proteins) were applied for western blot. Quantitation of acetylated SMC3, SMC3 and tubulin proteins among total input proteins. The intensity of each band was quantified by Quantity One (Bio-Rad) and plotted to validate that the protein levels are proportional to the total inputs within the range tested. (B) Over-expression of CRL4 subunits is able to partially restore the levels of Smc3ac caused by ESCO2 depletion. The representative immunoblots (upper) along with the relative SMC3ac levels of three experiments (lower) are shown. SMC3ac stands for acetylated SMC3. The statistical significance was calculated via student’s t-test. (C-E) Representative biological repeats of Fig 5A. (TIF) [file pgen.1007685.s005.tif]
